# Supplementary material for: Novel Chain-End Modification of Polymer Iodides via Reversible Complexation-Mediated Polymerization with Functionalized Radical Generation Agents
Source: Polymers (Basel). 2023 Jun 13;15(12):2667. doi: 10.3390/polym15122667 (PMC10305694; doi:10.3390/polym15122667)
Supplement: Supplementary file 1 [file polymers-15-02667-s001.zip › polymers-2391962-supplementary.pdf]

polymers

MDPI

Supplementary Materials: Novel Chain-End Modification of Polymer Iodides via Reversible Complexation-Mediated Polymerization with Functionalized Radical Generation Agents

Kazuya Ohtani [1], Kanta Shimizu [1], Tatsuhiro Takahashi [1] and Masumi Takamura [2,*]

[1] Department of Organic Materials Science, Graduated School of Organic Materials Science, Yamagata University, 4-3-16 Jonan, Yonezawa 992-8510, Japan; t221002m@st.yamagata-u.ac.jp (K.O.); smknt19981007@gmail.com (K.S.); effort@yz.yamagata-u.ac.jp (T.T.)

[2] Yamagata University Inkjet Development Center, 1- 808-48 Arcadia, Yonezawa 992-0119, Japan

* Correspondence: masumi_takamura@yz.yamagata-u.ac.jp

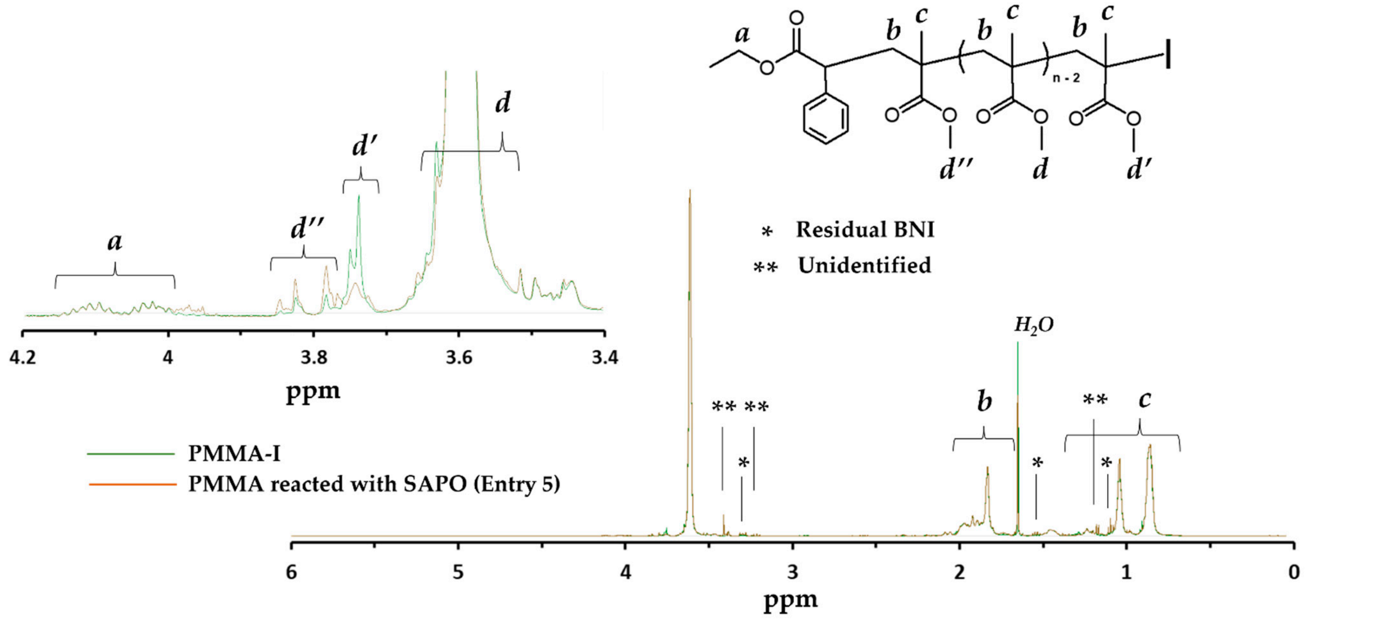

Figure S1. ^{1}H-NMR spectra of PMMA modified with SAPO (Entry 5) and PMMA-I (precursor).

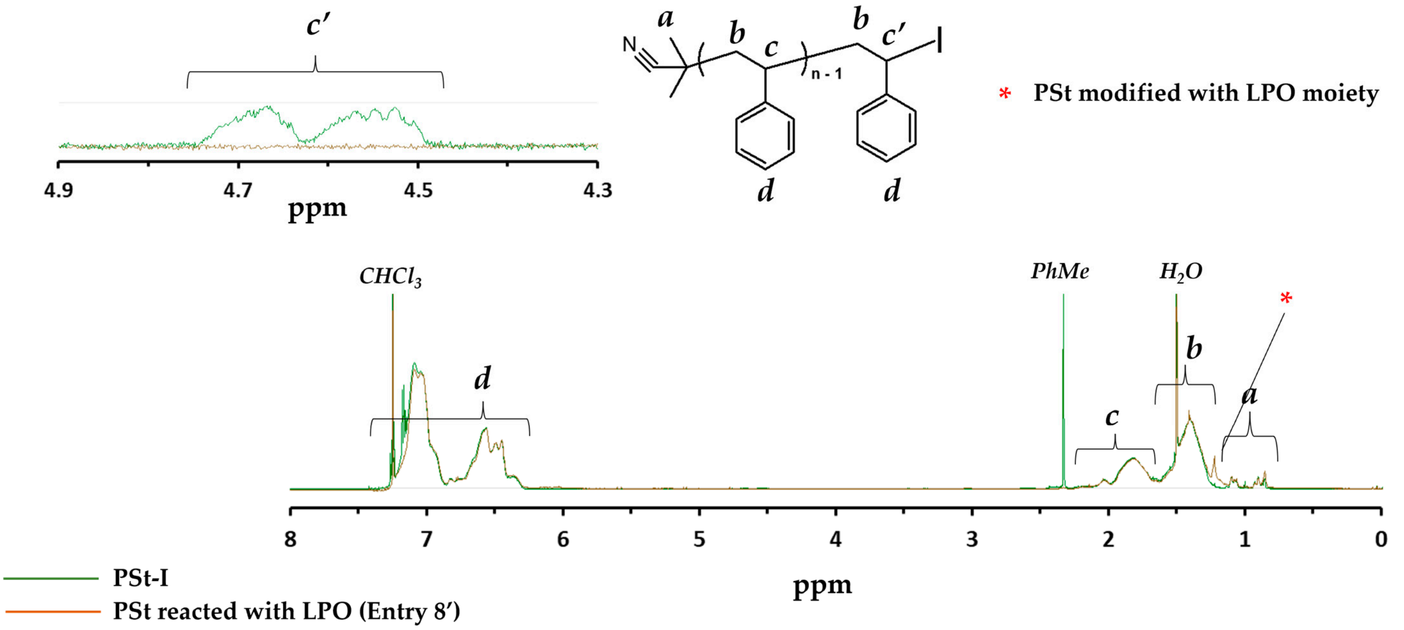

Figure S2. ^{1}H-NMR spectra of PSt modified with LPO (Entry 8') and PSt-I (precursor).

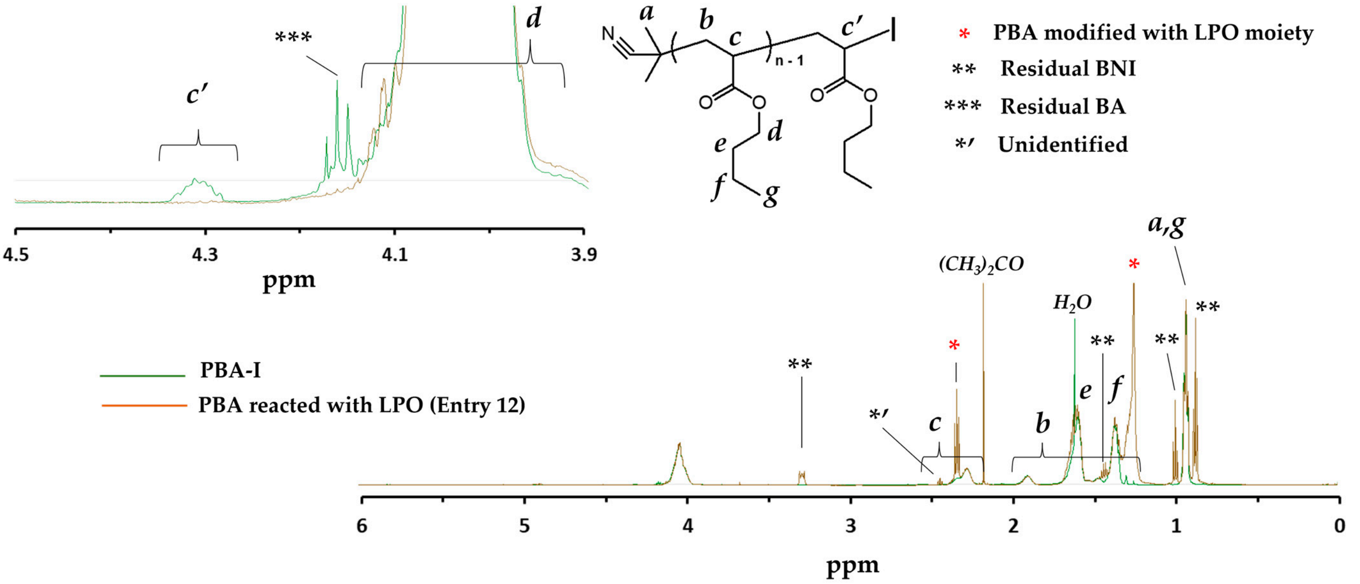

Figure S3. ^{1}H-NMR spectra of PBA modified with LPO (Entry 12) and PBA-I (precursor).

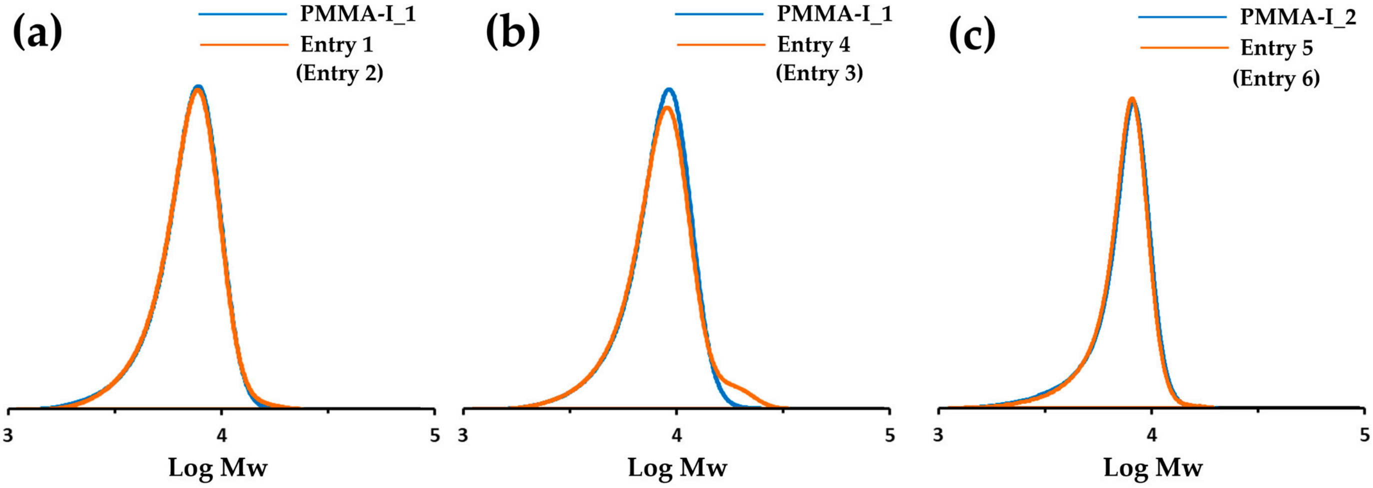

Figure S4. SEC chromatograms before and after chain-end modification for (a) Entry 1, (b) Entry 4, and (c) Entry 5.

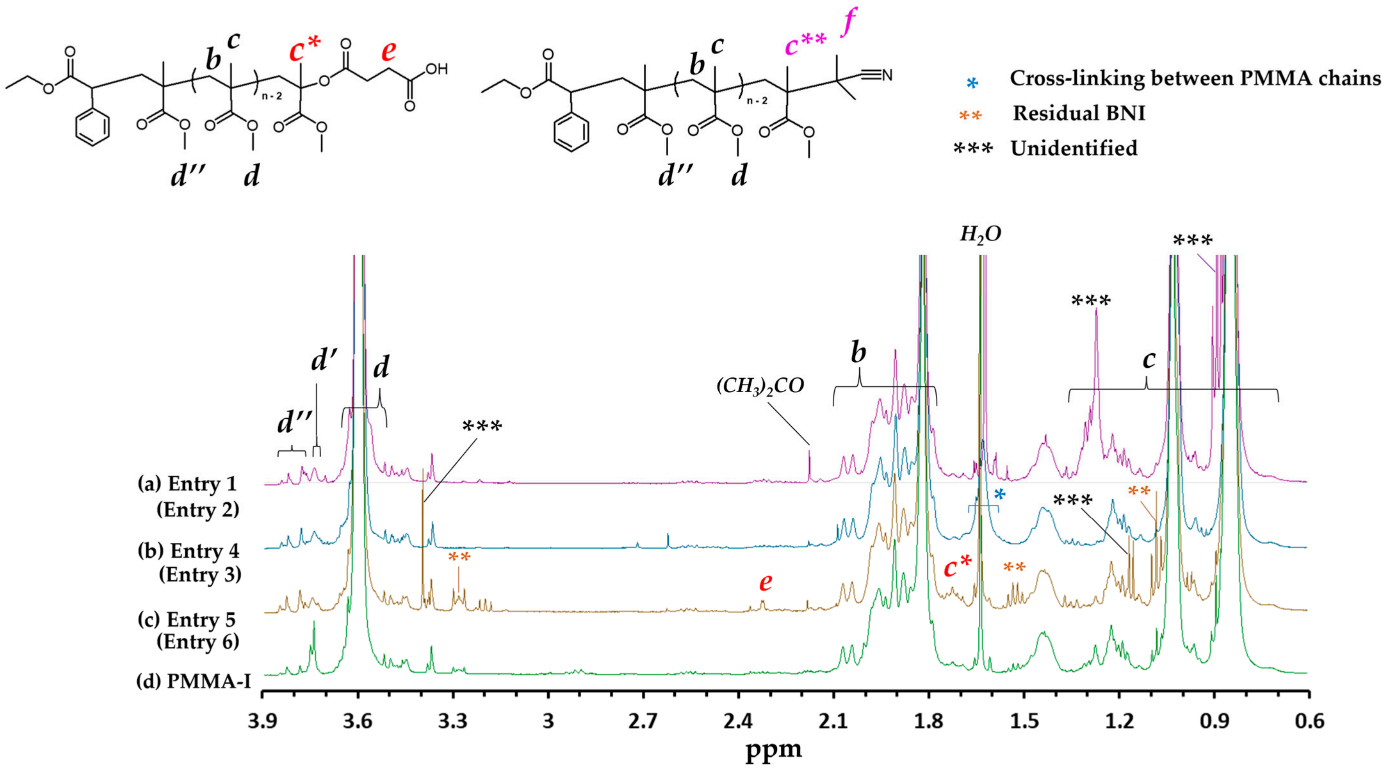

Figure S5. ^{1}H-NMR spectra of PMMAs modified with (**a**) only azo compound AIBN (Entry 1), (**b**) only diacyl peroxide SAPO (Entry 4), (**c**) combination of SAPO and iodine abstraction catalyst BNI (Entry 5), and (**d**) precursor PMMA-I.

Table S1. Combination ratio for radicals (R·) produced by decomposition of radical generating agent (R-R) to PhE-I radical (PhE·).

Radical generating agent (R-R)			Combination ratio between R·and PhE [%] [a]
**Type**	**Abbrev.**	**Structure of generated radical (R·)**	
Azo compound	AIBN	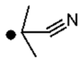	18
Diacyl peroxide	LPO	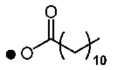	100
	BPO	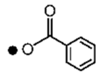	100

[a] combination ratio for R· and PhE· was calculated by integrating the peak corresponding to PhE-R generated by combination of PhE· and R

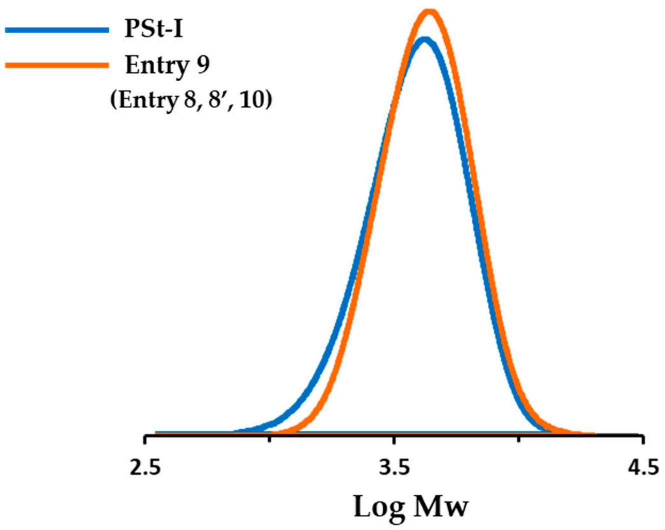

Figure S6. SEC chromatograms before and after chain-end modification for Entry 9.

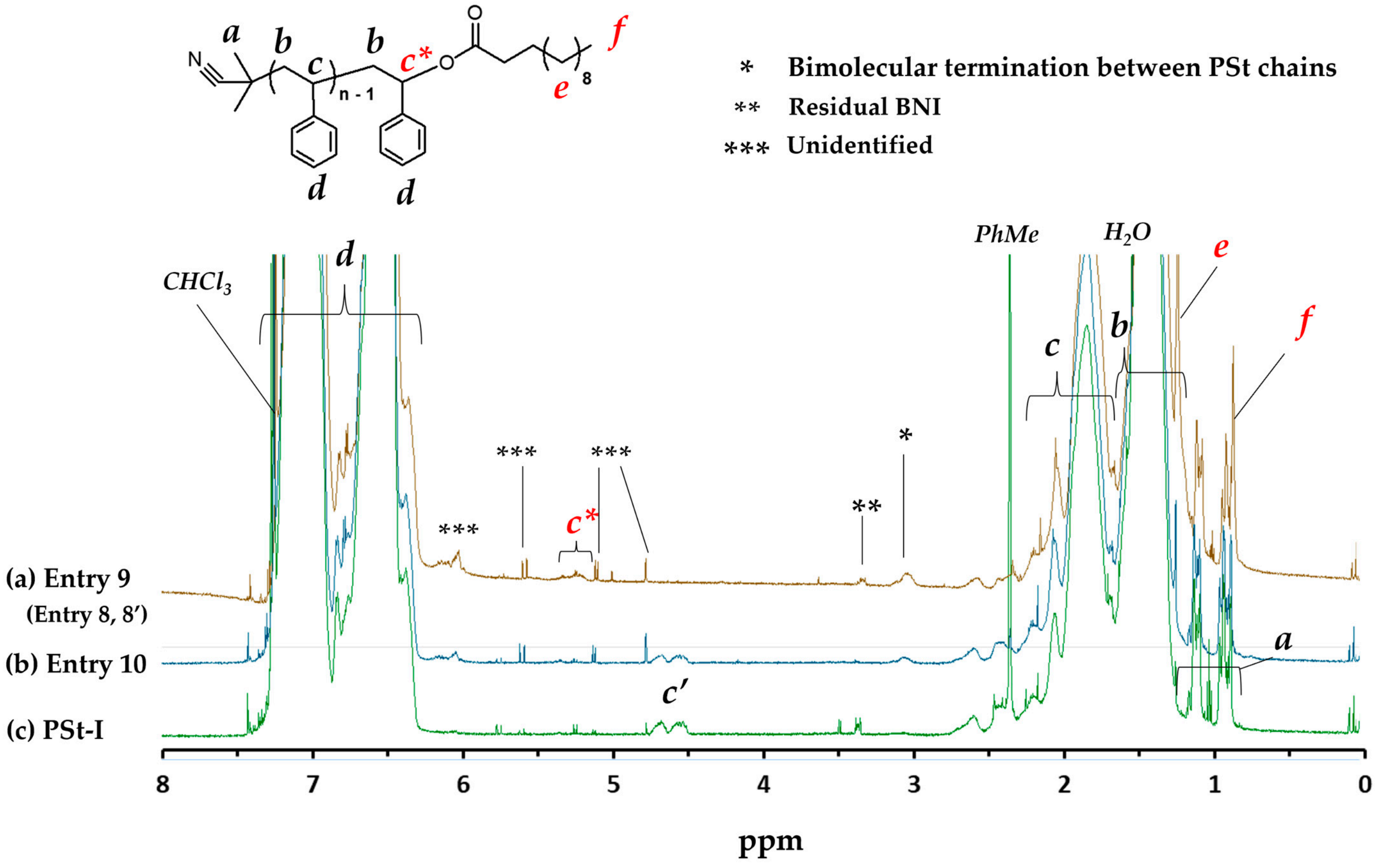

Figure S7. ^{1}H-NMR spectra of PSt modified with (**a**) BNBr (Entry 9), (**b**) BSI (Entry 10), and (c) precursor PSt-I.

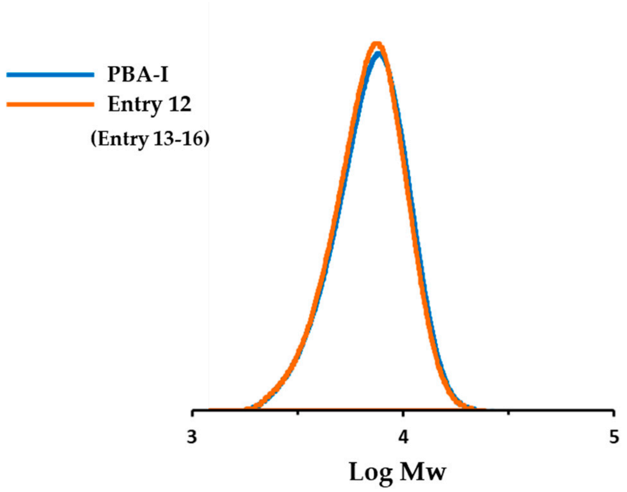

Figure S8. SEC chromatograms before and after chain-end modification for Entry 12.

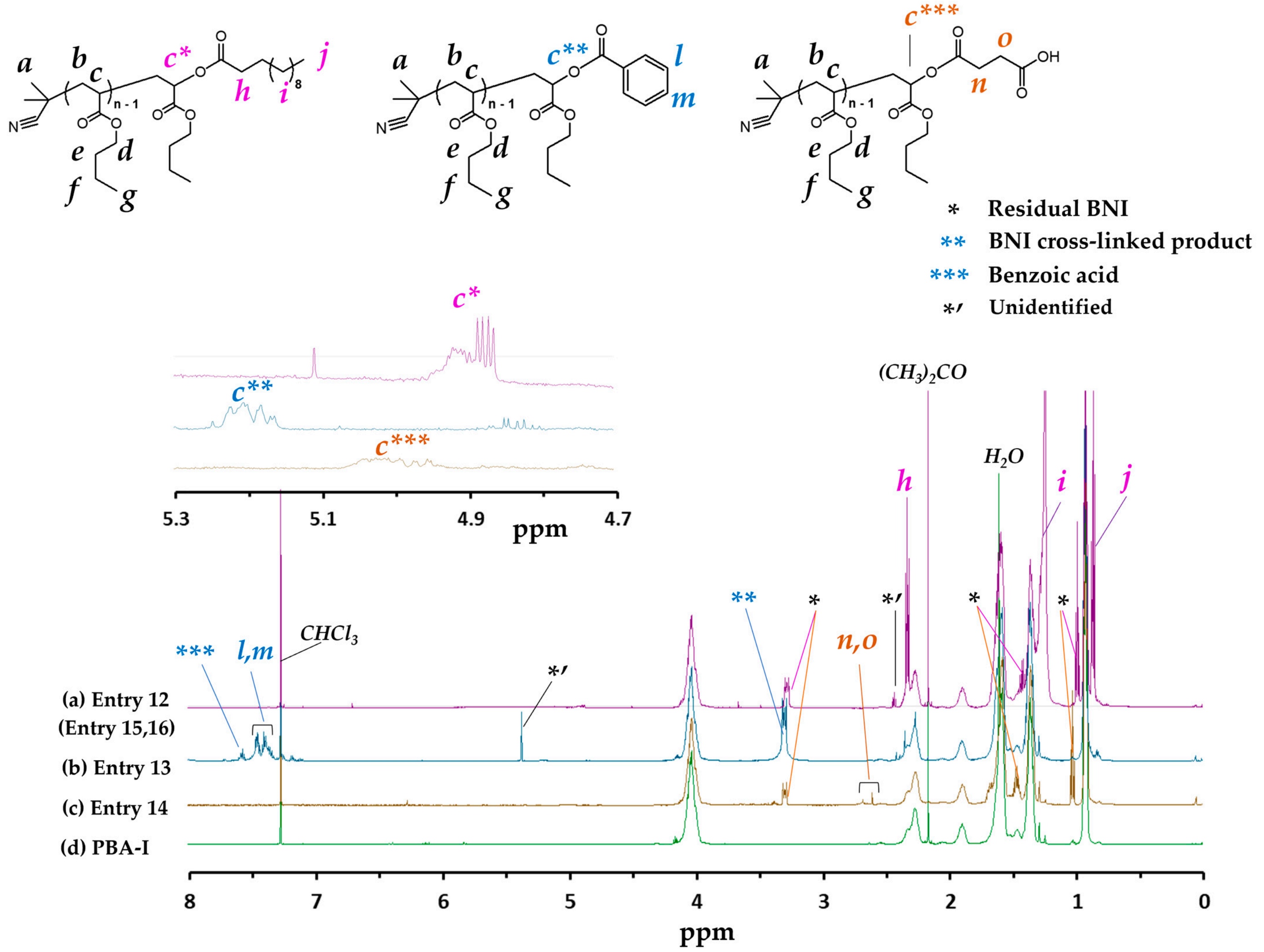

Figure S9. ^{1}H-NMR spectra of PBAs modified with different diacyl peroxides; (**a**) LPO (Entry 9), (**b**) BPO (Entry 13) and (**c**) SAPO (Entry 14), (**d**) precursor PBA-I.
